# Quantification by droplet digital PCR and species identification by metabarcoding of environmental (e)DNA from Blainville’s beaked whales, with assisted localization from an acoustic array

**DOI:** 10.1371/journal.pone.0291187

**Published:** 2023-09-13

**Authors:** Charles Scott Baker, Diane Claridge, Charlotte Dunn, Thomas Fetherston, Dorothy Nevé Baker, Holger Klinck, Debbie Steel

**Affiliations:** 1 Marine Mammal Institute, Hatfield Marine Science Center, Oregon State University, Newport, OR, United States of America; 2 Bahamas Marine Mammal Research Organisation, Sandy Point, Abaco, The Bahamas; 3 Naval Undersea Warfare Center, Newport, RI, United States of America; 4 Department of Ecology and Evolutionary Biology, University of California, Santa Cruz, CA, United States of America; 5 Center for Conservation Bioacoustics Cornell Lab of Ornithology, Cornell University, Ithaca, NY, United States of America; Universita degli Studi della Tuscia, ITALY

## Abstract

Detection and identification of species, subspecies or stocks of whales, dolphins and porpoises at sea remain challenging, particularly for cryptic or elusive species like beaked whales (Family: Ziphiidae). Here we investigated the potential for using an acoustically assisted sampling design to collect environmental (e)DNA from beaked whales on the U.S. Navy’s Atlantic Undersea Test and Evaluation Center (AUTEC) in The Bahamas. During 12 days of August 2019, we conducted 9 small-boat surveys and collected 56 samples of seawater (paired subsamples of 1L each, including controls) using both a spatial collection design in the absence of visual confirmation of whales, and a serial collection design in the proximity of whales at the surface. There were 7 sightings of whales, including 11 Blainville’s beaked whales (*Mesoplodon densirostris*). All whales were located initially with the assistance of information from a bottom-mounted acoustic array available on the AUTEC range. Quantification by droplet digital (dd)PCR from the four spatial design collections showed no samples of eDNA above the threshold of detection and none of these 20 samples yielded amplicons for conventional or next-generation sequencing. Quantification of the 31 samples from four serial collections identified 11 likely positive detections. eDNA barcoding by conventional sequencing and eDNA metabarcoding by next-generation sequencing confirmed species identification for 9 samples from three of the four serial collections. We further resolved five intra-specific variants (i.e., haplotypes), two of which showed an exact match to previously published haplotypes and three that have not been reported previously to the international repository, GenBank. A minimum spanning network of the five eDNA haplotypes, with all other published haplotypes of Blainville’s beaked whales, suggested the potential for further resolution of differences between oceanic populations.

## Introduction

DNA barcoding is now a well-accepted tool in the taxonomy and conservation of cetaceans [1], providing for confident identification of species and, in some cases, delimitation of stocks, Distinct Population Segment or subspecies [2]. However, genetic sampling for species identification of some whales, dolphins and porpoises (cetaceans) at sea remains challenging. Most samples have been collected using a biopsy dart [3]. This requires a close approach of a vessel, usually to within a few 10s of meters, while the whale or dolphin is at the surface. It can also be limiting because of access, distribution or behavior of cetaceans. Some species are rare, cryptic or both, e.g., the beaked whales [4]. Others species are difficult to approach because of their elusive behavior, e.g., the pygmy and dwarf sperm whale (*Kogia* spp,) [5]. Finally, some species are considered vulnerable to disturbance from the close approach of a vessel or the biopsy sample itself [6].

Advances in analyses of environmental (e)DNA offer an alternative for detection and identification of rare, cryptic or vulnerable cetacean species [7, 8]. Here the DNA that is shed or excreted from individuals during normal activity can be collected from the environment, concentrated by filtering, and amplified via the Polymerase Chain Reaction (PCR) using primers targeted for specific taxonomic groups. eDNA has been used widely in freshwater systems [9–11], and is now finding a growing number of applications in the marine environment [12], including detection and identification of marine megafauna [13, 14]. Whales, dolphins and porpoises are potentially good candidates for eDNA sampling given their known tendency to release cellular DNA in shed skin, fecal plumes and the spout or “blow” [15, 16].

The methodology for eDNA sampling is advancing rapidly, as the number and range of applications are increasing. One of these advances is droplet-digital (dd)PCR, a technology for quantifying low levels of DNA by fractionating a PCR reaction into more than 20,000 droplets using an oil emulsion [17]. Amplification of the target DNA is quantified by incorporating a fluorescent dye into a molecular probe designed to target a specific sequence bracketed by the PCR primers. The target-positive and target-negative droplets are individually counted by passing them through a fluorescence detector, similar to a flow cytometer. The ratio of the target-positive to the target-negative droplets is used to estimate the number of copies of the target DNA in the sample. Thus, unlike conventional quantitative (q)PCR, ddPCR allows for direct quantification without the need for standard curves, eliminating the variance associated with creating standards with each run [18]. Owing to reaction partitioning, ddPCR is also thought to show an increased tolerance to inhibitors, making it an attractive alternative to qPCR for detection and quantification of eDNA [19].

Another advance in technology is next-generation sequencing (NGS) for eDNA metabarcoding [20]. With instruments such as the Illumina MiSeq or HiSeq, it is possible to sequence many millions of short reads (usually 250 base pairs or less in length) and to index multiple samples in a single run. The short reads can then be processed with a bioinformatic pipeline to sort through the diversity of Amplicon Sequence Variants (ASVs) from a multiplex of eDNA samples (e.g., [21]). Using so-called “universal primers” to amplify conserved regions of the mitochondrial genome, it is possible to survey the diversity of species or Operational Taxonomic Units from an entire ecological community. Alternatively, taxon-specific primers can be used to amplify more variable regions of the mitochondrial genome, providing information on intra-specific diversity or mtDNA haplotypes. Although eDNA barcoding remains a useful tool for single-species identification [7], conventional methods for sequencing cannot distinguish multiple species or resolve multiple amplicon sequence variants (ASVs or haplotypes), from a mixed sample.

Here, we evaluate the potential for an acoustically assisted sampling of eDNA from Blainville’s beaked whales (*Mesoplodon densirostris*) in the waters of the U.S. Navy’s Atlantic Undersea Test and Evaluation Center (AUTEC), in The Bahamas. For this, we took advantage of the cabled array of fixed hydrophones to provide real-time, automated detection and localization of cetaceans [22, 23]. Using the acoustic localization, we collected seawater from the proximity of whales sighted at the surface and at the approximate position of whales located acoustically at depths. We continued to refine laboratory methods for eDNA extraction and quantification by ddPCR and to improve confidence in the identification of variants by requiring a majority rule in triplicate re-sequencing. For the former, we validated the limits of ddPCR detection with conventional sequencing for DNA barcoding and NGS for eDNA metabarcoding. Unlike some eDNA studies, which rely on qPCR for both quantification and species identification, our protocol involves, first, quantification by ddPCR and, second, identification of species and haplotypes by conventional and NGS sequencing.

## Materials and methods

### AUTEC field effort and sampling design

Samples of seawater for environment (e)DNA were conducted during small-boat surveys on the AUTEC range, Andros Island, in The Bahamas, from 31 July to 13 August 2019. The real-time acoustic localization from the Marine Mammal Monitoring on Navy Ranges (M3R) program was used to direct the boat to the vicinity of acoustically active Blainville’s beaked whales. When the whales surfaced and were detected visually, the boat first moved into a position to photograph all individuals in the group. When the whales submerged, the boat moved into the position of the visible slick, or “fluke print”, to initiate a *serial* sampling design. After each surfacing interval, seawater samples were collected from the surface in a 1L, wide-mouth, sterile Nalgene bottle. All samples were collected in pairs, one from the starboard and one from the port side of the boat. Following a “terminal dive” (a foraging dive), the vessel was repositioned in the fluke print and samples were collected at the time of the dive and at 15-minute intervals for up to two hours. If the location of the whale could not be visually confirmed, the boat was moved to the best approximation from the acoustic detection. We then initiated a localized *spatial* sampling series. For this, paired seawater samples (2 x 1L subsamples) were collected at 100 m intervals for 400 m along both north-south and east-west axes (i.e., 9 paired samples), intersecting at the primary coordinates from the acoustic localization. The shipboard GPS was used to position the boat for the prescribed distances.

### M3R system for acoustic detection and localization

The M3R program has developed a system to allow real-time, automated detection, classification, and localization of marine mammals on the U.S. Navy’s undersea test and training ranges [22, 23]. The AUTEC M3R system consists of the M3R cluster and three monitoring stations. The cluster records the raw data, runs the detection/classification, data association and localization software, and then sends it to the display machines in the monitoring stations. The display used during the eDNA sampling was M3RWorldView, based upon NASA’s open-source World Wind program with marine mammal detection software overlaid (see S1 Fig). This software overlay characterizes click detections by frequency band to identify species, e.g., vocalization of Blainville’s beaked whale (*Mesoplodon densirostris*) characteristically show acoustic energy above 20 kHz [24]. M3R software computes localizations from time difference of arrival data using a hyperbolic, multi-lateration algorithm, providing position data [25].

### Standardization of eDNA collection, filtering and extraction

Seawater samples were collected in paired subsamples of 1L each and stored on ice until returning to the AUTEC housing at the end of each day. Both subsamples were then filtered, on site, through a 0.4 micron, polycarbonate track-etched filter (PCTE, GE Lifesciences, USA) using a portable Nalgene™ filter unit and low-pressure vacuum pump. The sample processing included 4 seawater blanks as controls. The filters were stored in 1mL of Longmire’s solution for transport back to a ‘clean room’ at the Hatfield Marine Science Center, Oregon State University (OSU). All sample bottles and filter units were decontaminated by soaking in 10% bleach, overnight, and rinsing in tap water before re-use.

On return to the laboratory, one half of the Longmire’s solution was archived. The remaining Longmire’s solution and the filter were extracted by a conventional phenol/chloroform/isoamyl alcohol (PCI) method [7]. The PCI extraction was chosen because chloroform dissolves the PCTE filters, optimizing the recovery of the eDNA [26]. Initial efforts to amplify eDNA showed evidence of inhibition to conventional PCR, presumably resulting from co-extraction of biological compounds from the surface seawater samples. PCR inhibitors were removed or reduced using the OneStep^TM^ PCR Inhibitor Removal Kit (Zymo Research). Following the OneStep cleanup, all samples were standardized to approximately 50μl volume in TE buffer, i.e., the eDNA extracted from a 1L subsample was recovered in a final volume of 50μl.

### eDNA barcoding primers, conventional PCR and sequencing

A comprehensive reference database representing mitochondrial (mt) DNA control region sequences of most known cetacean species is available through a web-based program, www.dna-surveillance.auckland.ac.nz [27]. Using this validated database, we chose two primer pairs to amplify and sequence an overlapping fragment of the cetacean mtDNA control region or d-loop (Fig 2). The primers Dlp1.5 to Dlp5 amplify a fragment of about 530 base pairs (bp) in length and primers Dlp1.5 to Dlp4 amplify a fragment of 390 bp, nested within Dlp1.5 to 5 [4]. The sequence length of either combination of primers is sufficient for confident identification of cetacean species, and in some cases identification geographic variants (i.e., haplotypes), while allowing for amplification of degraded eDNA [4]. These primers and the reference database of control region sequences have been used extensively for the species identification of whale-meat products sold in fisheries markets of Japan and South Korea [1, 28].

We attempted to amplify the eDNA of each sample using both primer pairs, Dlp1.5 to 5 and 1.5 to 4 (nested). For this 1μl of the eDNA extraction was added to 20μl of standard PCR reaction (Platinum *Taq*), following standard conditions described in Baker et al. [7]. The success of the conventional PCR was judged by the visualization of the amplicon on an agarose gel. All visible amplicons were sequenced in both directions with Big Dye terminator chemistry (ThermoFisher Scientific, USA) on an ABI3730xl. Sequences were aligned to known haplotypes of Blainville’s whales [4, 29] and visually inspected with the software Sequencher 4.1 (Gene Code).

### ddPCR, DNA barcoding and metabarcoding

**Fig 1:**
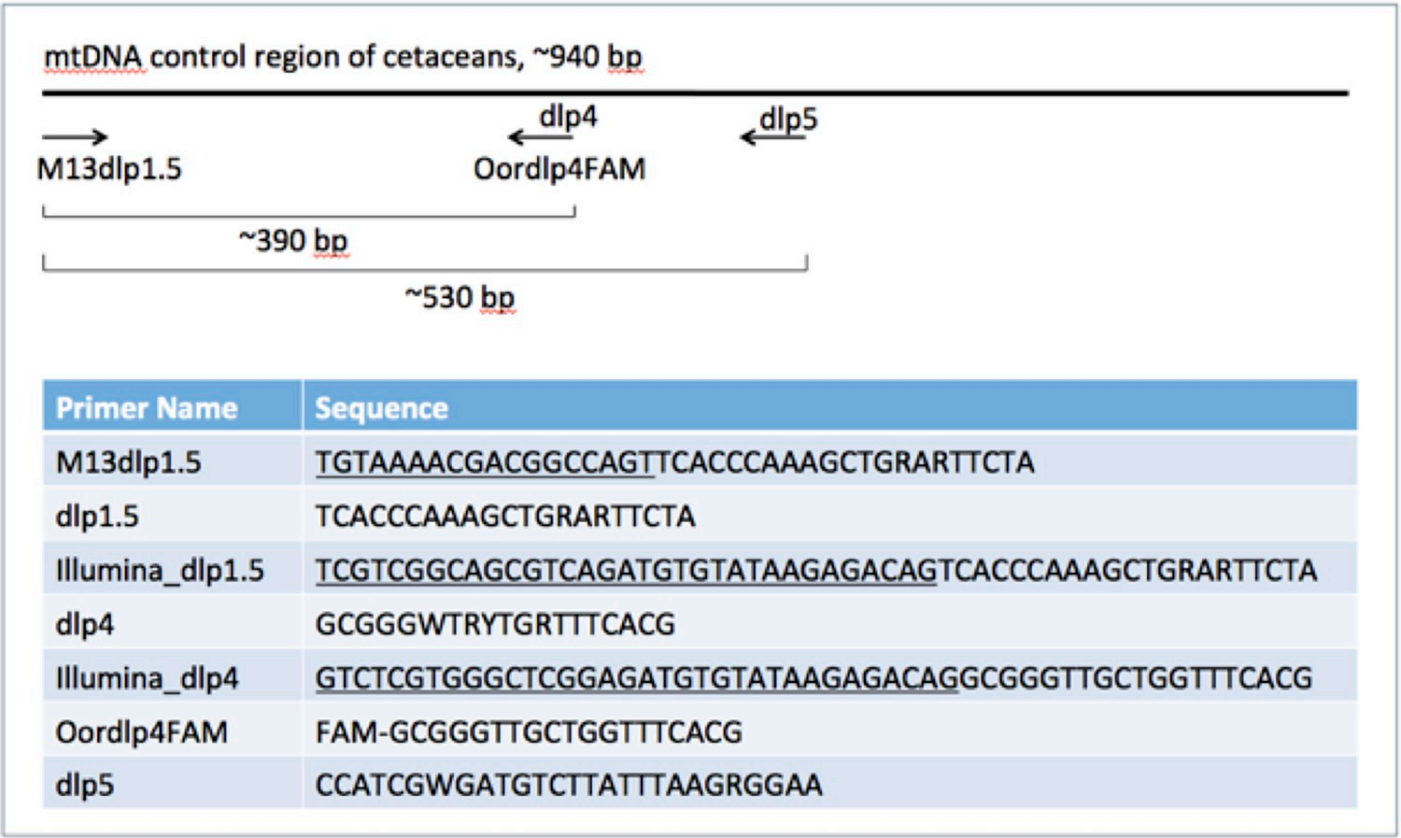
Primer sequences used in eDNA barcoding and metabarcoding and approximate locations on the control region of the cetacean mtDNA. Some taxon-base primers were modified to include adaptors used in library preparation or attachment of fluorescent probe (FAM), shown as underline.

The extracted eDNA was quantified by droplet digital (dd)PCR using the BioRad QX200 Droplet Digital™ instrument and a ddPCR^TM^ Supermix for Residual DNA Quantification (BioRad). For this we used the primers Dlp1.5 to 5 with a molecular probe designed from the nested primer, Oordlp4FAM (Fig 1, [7]). The ddPCR amplification of each sample was run in triplicate. Two of the three replicates were used for ddPCR quantification with the droplet reader. The target-positive and target-negative droplets of the ddPCR reactions were visualized and analyzed using the manufacturer’s software, QuantaSoft [30]. Quantification of target DNA, in copies/μL of reaction is based on an assumption of a Poisson distribution of the target DNA among the more than 20,000 droplets from a typical 20 μL reaction. The third ddPCR reaction was set aside for re-amplification by conventional PCR and Sanger sequencing. After ddPCR amplification, samples showing a likely positive detection (i.e., a relaxed threshold of approximately 0.1 copies/μl, see [7]) were harvested from the oil emulsion and reamplified by conventional PCR. This two-step amplification protocol was intended to help overcome the potential for inhibitors in the conventional PCR.

For eDNA metabarcoding on the Illumina MiSeq, we used a high-fidelity polymerase (Kapa Hifi, Roche) to first amplify the Dlp1.5 to Dlp5 fragment from the eDNA extractions using 20x cycles. A second round of 20x amplification was then used with the Dlp1.5 primer and the nested Dlp4, both of which included the Illumina adaptors (Fig 1). The amplicon from each sample was then prepared for sequencing on an Illumina MiSeq using a Nano flowcell following a standard library preparation with indexing. The metabarcoding library was run as a multiplex of 48 samples with the expectation of >20,000 reads per sample using 250 bp, paired-end reads. All samples were repeated three times, with independent Illumina libraries, on independent sequencing runs, to identify and subsequently remove polymerase or sequencing artifacts. All ddPCR and MiSeq runs were conducted by the OSU core facility, the Center for Genome Research and Computing (CGRB).

### Quality control and informatics

Conventional PCR and sequencing included the usual no-template control and visual review of electropherograms for identification of haplotypes and variants (e.g., [28]). The output of the MiSeq was de-multiplexed and the paired-end reads were assembled using the program Qiime2 [21]. The assembled reads, or ‘haplotypes’, were then submitted to a search of GenBank for initial species identification and to the curated database maintained on *DNA-surveillance* for confirmation [31]. Sequences arising from host-cell contamination of molecular reagents (e.g., mouse, pig and cow) were identified by Qiime2 and removed [32]. Within-species sequence variants (i.e., mtDNA haplotypes) of cetaceans were evaluated for processing or polymerase errors. We accepted a haplotype variant only if it was repeated in at least two independent runs of the MiSeq (i.e., a majority rules criterion), or replicated by conventional PCR and sequencing. The relationship of the eDNA variants to the available haplotypes from GenBank was reconstructed using a minimum spanning network, as implemented in the program POPART [33].

## Results

### Vessel surveys and photo-identification

We conducted 9 small-boat surveys, covering 508 km of survey tracks during a total of 51 hours on the water. There were 7 cetacean sightings, including 5 groups of Blainville’s beaked whales. Group sizes ranged from 1–3 whales, and included a total of 11 individuals. Of these, ten whales were individually identified from high-quality photographs, including resightings of four whales previously photo-identified on the AUTEC range. Two of the known individuals had been sampled previously with a biopsy dart. One new individual was sampled with a biopsy dart during this field effort. Photo-identification codes and notes on individual resighting records are included in S1 Table.

### Acoustic localization and sample collection

**Fig 2:**
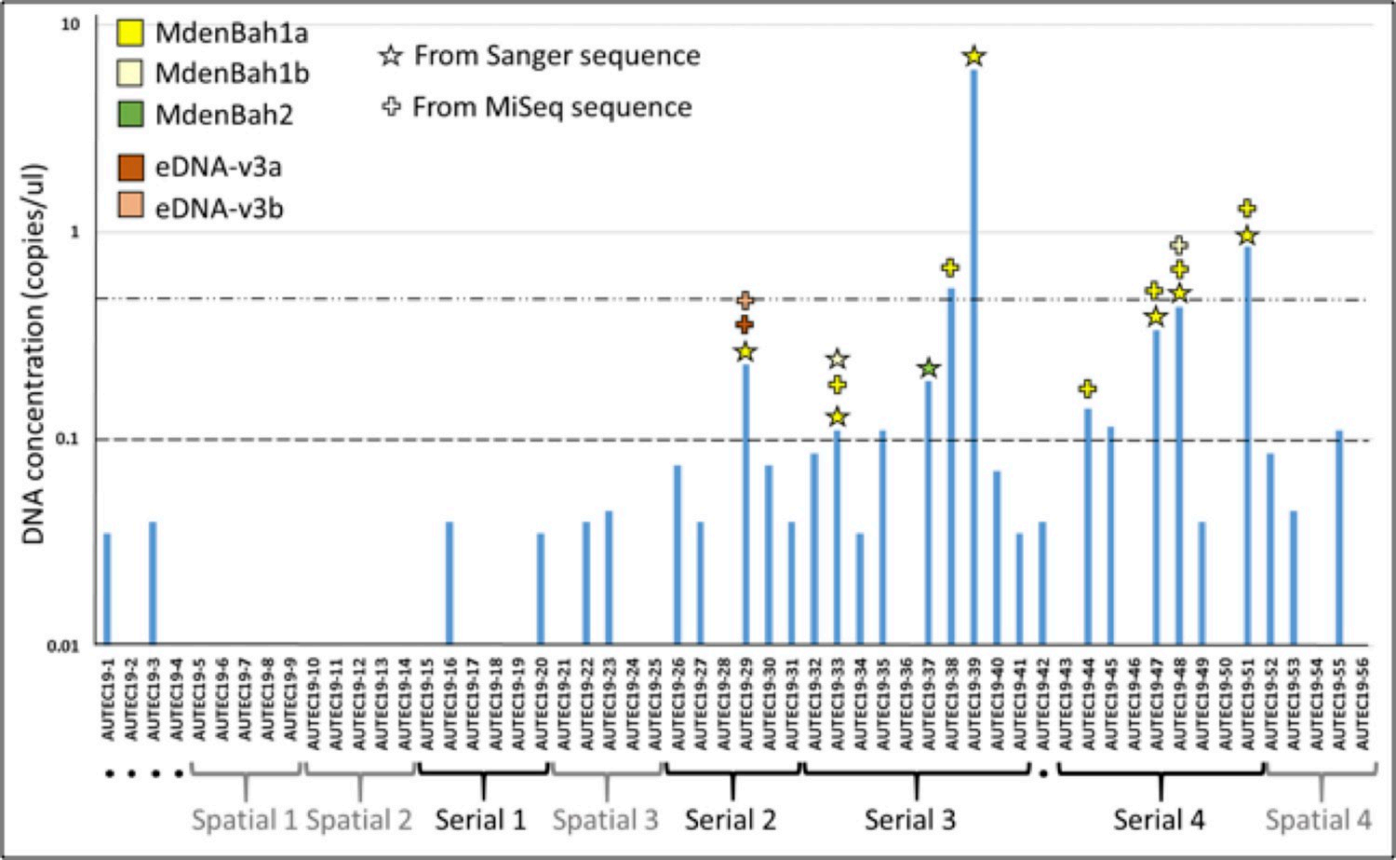
Average DNA concentration in copies/ul as determined by droplet digital (dd) PCR (run in duplicate). The dash/dot horizontal line represents a concentration of 0.5 copies/ul, previously determined to be a strict threshold (i.e., high probability) for a subsequent positive detection by eDNA barcoding [7]. The dashed line represents a concentration of approximately 0.1 copies/ul, previously considered to be a relaxed threshold for a likely positive detection. Stars represent samples that were identified as *Mesoplodon densirostris* via conventional eDNA barcoding and crosses represent samples identified via MiSeq. The colors refer to the sequence variant identified (i.e., mtDNA haplotypes).

All whales were located initially with the assistance of localization from the acoustic array (see S1 Fig). However, as beaked whales vocalize primarily while foraging at depths [34], the M3R operator cannot provide a precise position for the whales as they ascend, or once they reach the surface. Despite this limitation, we collected 56 paired samples of 1L each (i.e., 102 L total) within the vicinity of beaked whales. This included 4 spatial design collections (20 paired samples total) in the absence of any visible proximity to the whales, 4 serial design collections (31 paired samples total) in the visible proximity of the whales and 5 opportunistic samples in the acoustic proximity of the whales (Fig 2). An additional 12 samples were included in relevant analyses as positive and negative controls for either field or laboratory handling (see S1 Table).

### Conventional PCR and inhibitors

Following the PCI extraction, all 56 samples were tested for inhibitors by convention PCR, using a two-stage, nested amplification protocol with Dlp1.5 to Dlp5, followed by Dlp1.5 to Dlp4. No samples yielded a visible amplicon, suggesting either inhibitors to the PCR reaction or very low concentrations of eDNA in the samples, or both.

### ddPCR quantification and conventional sequencing of eDNA barcodes

Following clean-up of inhibitors with the OneStep^TM^ PCR Inhibitor Removal Kit (Zymo Research), all samples were submitted for ddPCR in triplicate, using the primer Oordlp4FAM as a florescent probe. The average values of the ddPCR, run in duplicate, were low for most samples, with many having 95% confidence limits overlapping with zero (see S1 Table). Only 12 of the 56 samples exceeded the relaxed threshold of approximately 0.1 copies/μl, considered a likely positive for detection, and only three of these met the strict threshold of 0.5 copies/μl (see [7]). Only one of the 12 likely positive samples was collected in a spatial design. Most of the other samples collected in the four spatial designs showed values of 0.0 copies/μl (Fig 2).

A third run of the ddPCR was reserved from the droplet counter and used, instead, to harvest amplifiable products for subsequent conventional PCR and sequencing. This protocol was intended to help overcome any residual inhibitors and to confirm species identification by conventional eDNA barcoding. Of the 12 likely positive samples, as quantified by ddPCR, 7 yielded amplicons suitable for conventional sequencing. These 7 sequences confirmed species identification of Blainville’s beaked whale and resolved two sequence variants, i.e., haplotypes. A search of GenBank confirmed identity with two mtDNA haplotypes reported previously from the Atlantic Ocean, referred to here as MdenBah1a and MdenBah2 (Table 1; [29]).

**Table 1:** The variable sites resolving by eDNA barcodes (i.e., mtDNA haplotypes) from Blainville’s beaked whales on the AUTEC range compared to those available on the international repository, GenBank. Note that trimming the length of the reference sequences to the fragment identified in the eDNA barcodes (317bp) resulted in collapse of some variant represented in GenBank. Match-to-first shows the reference sequence on the first line and a period (.) to indicate identity. An insertion/deletion is considered a fifth character and indicated by two dashes (--). Sequences of the 5 haplotypes reported here have been submitted to GenBank (as a PopSet, GenBank Accession Numbers MW526241-MW526245).

GenBank #	Haplotype Code	48	77	78	81	107	108	120	127	133	149	265	287	293
KF032867/MW526242	MdenBah1a	A	A	T	T	A	G	C	G	A	G	C	G	G
MW526245	MdenBah1b	.	--	.	.	.	.	.	.	.	.	.	.	.
KF032874	MdenBah3	.	.	.	.	.	.	.	.	.	.	T	.	.
AB610396	Mauritius	T	.	.	.	.	.	.	.	.	.	T	.	.
AB610397	Mauritius	T	.	.	.	.	R	.	.	.	.	T	.	.
KF032873/MW526241	MdenBah2	.	.	C	C	.	.	.	.	.	.	T	.	.
AY579513	MdeNZ1	.	.	.	.	.	.	.	.	G	.	T	.	.
KF032862	MdenHI2	.	.	.	.	.	.	.	C	G	A	T	.	.
KF032860	MdenSBCA	.	.	.	.	G	.	.	.	G	.	.	.	.
KY542115	China	.	.	.	.	.	.	.	.	G	.	.	.	.
KC540691	Kiribati002	.	.	.	.	.	.	.	.	G	.	.	A	A
KC540694	Kiribati018	.	.	.	.	.	.	.	.	G	.	.	A	.
KF032861	MdenstrandNZ	.	.	.	.	.	.	.	C	G	.	.	.	.
MW526243	eDNAv3a	.	.	.	.	.	A	.	.	.	.	.	.	.
MW526244	eDNAv3b	.	--	.	.	.	A	.	.	.	.	.	.	.

### eDNA metabarcoding and novel haplotype variants

To assess the potential to detect further haplotype variants with next-generation sequencing, we submitted the 12 likely positive samples for eDNA metabarcoding on the Illumina MiSeq as both a series of individual and pooled libraries. To reduce sequencing errors, we used a high-fidelity polymerase (Kapa HiFi) for amplification of the primary eDNA extractions (i.e., not from the ddPCR reactions). As a further validation of quality control, all PCR reactions, Illumina libraries and MiSeq runs were repeated three times. Of the 12 samples submitted, 7 provided sequences identified as Blainville’s beaked whale, including two samples that had not provided results from amplification of the ddPCR reaction for conventional sequencing (Table 2).

**Table 2:** Summary of the five haplotypes (317bp in length) identified in 9 of the 12 samples judged to be likely positives from ddPCR quantification for eDNA from Blainville’s beaked whales. Sample codes correspond to Fig 2 and S1 Table. ddPCR concentrations are in copies/μl. Haplotypes identified by conventional amplification and Sanger sequencing are indicated by a ‘+’. Haplotypes identified by next-generation sequencing are represented by the number of assembled reads from triplicate libraries and runs of the MiSeq (250 bp, paired-end). Three of the candidate samples, indicated by an ‘*’, failed to provide an identifiable sequence by either method. See Table 1 for sequence variation defining each haplotype.

Haplotypes	AUTEC19 sample codes											
	#29	#33	#35*	#37	#38	#39	#44	#45*	#47	#48	#51	#55*
MdenBah1a	+	9387+			61	+	127		1224+	1069+	44+	
MdenBah1b		+								183		
MdenBah2				+								
eDNAv3a	126											
eDNAv3b	3011											

**Fig 3:**
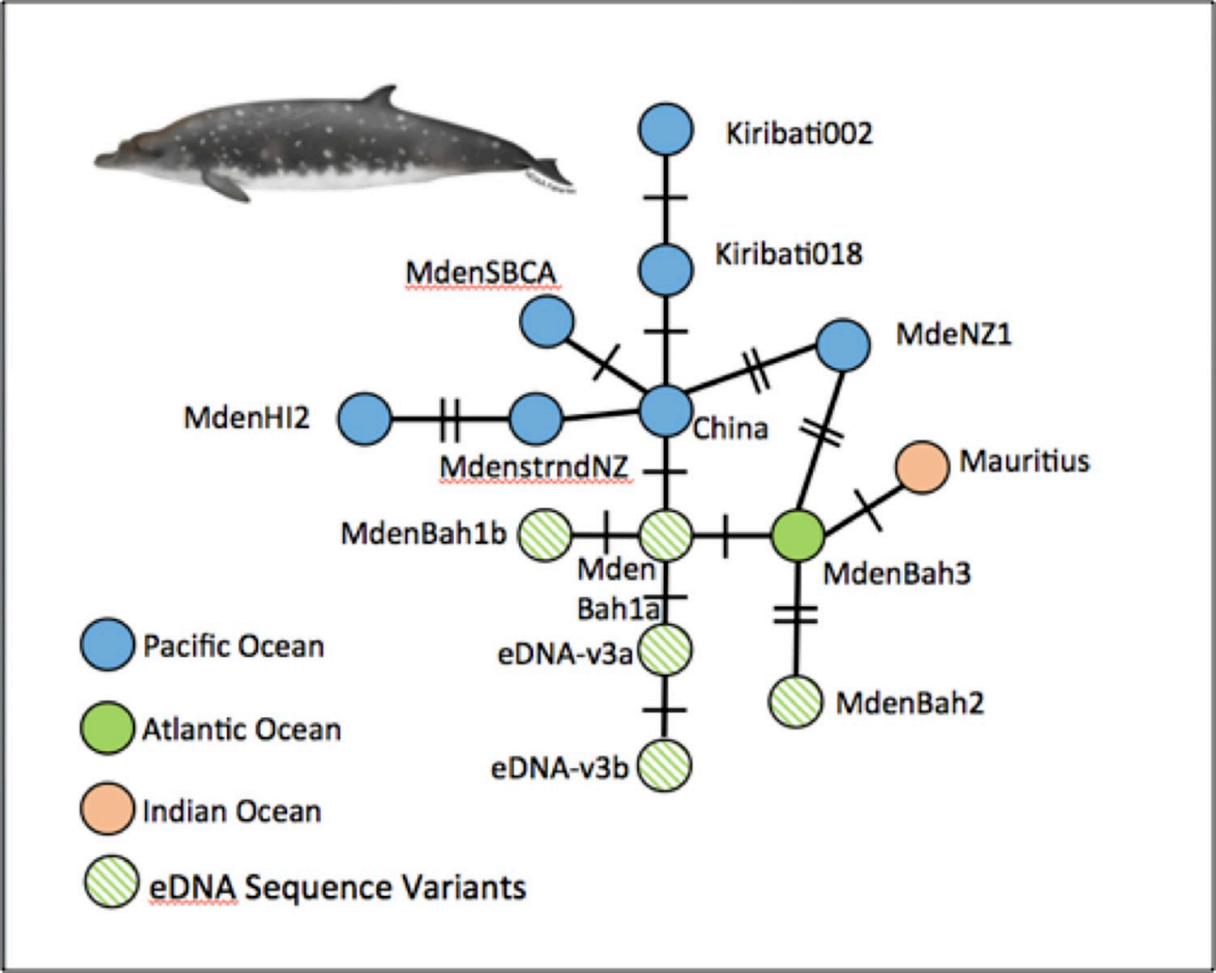
The relationships of mtDNA control region haplotypes of Blainville’s beaked whales from available public sources (e.g., GenBank) and the 5 haplotypes detected by eDNA barcoding and metabarcoding on the AUTEC range (see Table 2). The lines connect the most closely related haplotypes using a minimum spanning network [33]. The short, perpendicular lines indicate the number of nucleotide differences or insertion/deletion events resolving the haplotypes.

Considering both the conventional sequencing and the MiSeq results, 9 of the 12 samples considered to be likely positives from the ddPCR provided species identification of Blainville’s beaked whales. A review of the sequencing variants provided evidence of 5 haplotypes among the 9 samples (Table 2). These 5 haplotypes were found in at least two independent runs of the MiSeq or supported by conventional PCR and sequencing. Two of the haplotypes, MdenBah1a and MdenBah2, discussed above, were an exact match to sequences reported previously from the North Atlantic and available on GenBank [29]. The other three haplotypes have not, to our knowledge, been reported previously. A minimum spanning network of the eDNA variants and all previously reported haplotypes suggests the potential for detection of population structure from haplotype identity, at least at the oceanic scale (Fig 3).

## Discussion

Our results confirm the power of conventional eDNA barcoding and metabarcoding to identify species and intra-specific diversity of beaked whales, when sampled in the proximity of whales at the surface [7, 35, 36]. Variation in the success of identification by conventional and next-generation sequencing was consistent with ddPCR quantification. Samples with estimated concentrations of less than 0.1 copies/μL were unlikely to provide an amplicon for conventional or NGS sequencing, presumably reflecting the heterogeneity of capturing eDNA at low copy numbers [37, 38]. Despite the limitations of sampling, the broader application of eDNA sampling promises to greatly enhance success with genetic sampling of beaked whales and other deep-diving species that are cryptic or sensitive to the approach of vessels. The characterization of intra-specific diversity, as represented by haplotype variants, also shows promise for resolving population structure, at least at the oceanic scale. Although haplotype diversity is low in Blainville’s beaked whales, it was notable that none of the eDNA variants from the Bahamas were identical to those reported previously in the Pacific or Indian Oceans (Fig 3).

Our results also confirm the utility of combining acoustic detection and localization to aid in the collection of eDNA for species and haplotype identification. The potential to locate the Blainville’s beaked whales, in this study, was dependent on the cabled acoustic array and real-time output of the M3RWorldView software. Although the availability of such fixed-location, real-time acoustic arrays is limited to a small number of Navy ranges, technological and computational advances promise to provide some degree of real-time localization from drifting or towed hydrophones (e.g., [39]). The absence of eDNA detection for the spatial sampling design was disappointing but not surprising. The precision of the acoustic localization was limited by the absence of vocalizations during the surfacing of the whales and visual localization was limited by the need for calm seas (typically, < Beaufort 2). Thus, we did not actually know where the whale(s) surfaced and so were unable to judge the true distance of the whale at the surface from the location chosen to initiate the spatial sampling. These limitations are likely to be common in efforts to survey eDNA of beaked whales and other rare species, without some assistance from acoustic or visual localization.

Future sampling of the AUTEC range, or other acoustic array systems, could include eDNA at depth for both the beaked whales and their prey species [40]. Such an at-depth sampling design would be greatly enhanced by acoustic localization of the whales in three dimensions [41]. With this capability, sampling could be directed to target the location of the whales at depth and include additional identification of prey species using “universal primers” for eDNA metabarcoding. These methods would provide additional information on habitat quality and whale distribution, building on results of recent hydro-acoustic surveys [42].

## Supporting information

S1 Fig An example of acoustic activity and localization of beaked whales on the AUTEC range, from August 8, 2019, taken as screenshots from M3RWorldView (courtesy, T. Fetherston, Naval Undersea Warfare Center). Acoustic activity is indicated by the color of the hydrophone, with red indicating highest activity. The insets show the time (x axis) and frequency in kHz (y axis) of vocalizations on selected hydrophones. Blue triangles indicate positions of vocalizing beaked whales, e.g., the vocalizations of Blainville’s beaked whale (*Mesoplodon densirostris*) characteristically show acoustic energy above 20 kHz [34]. A cluster of beaked whale positions near hydrophones 3, 4 and 6 was used to locate the whales at the surface and collect eDNA for the third serial sample collection (see Table 1).
(PDF)

S1 Table Summary of eDNA sampling with results of ddPCR and metabarcoding. All samples are 2L of seawater filtered through 0.4 micron polycarbonate filter, unless otherwise noted. Samples shaded in yellow were chosen for ddPCR sequencing and metabarcoding based on relaxed threshold of 0.1 copies/μl from ddPCR quantification.
(XLSX)
