# Supplementary material for: Quantification by droplet digital PCR and species identification by metabarcoding of environmental (e)DNA from Blainville’s beaked whales, with assisted localization from an acoustic array
Source: PLoS One. 2023 Sep 13;18(9):e0291187. doi: 10.1371/journal.pone.0291187 (PMC10499200; doi:10.1371/journal.pone.0291187)

Supplementary Material: S1 Fig

S1 Fig. An example of acoustic activity and localization of beaked whales on the AUTEC range, from August 8, 2019, taken as screenshots from M3RWorldView (courtesy, T. Fetherston, Naval Undersea Warfare Center). Acoustic activity is indicated by the color of the hydrophone, with red indicating highest activity. The insets show the time (x axis) and frequency in kHz (y axis) of vocalizations on selected hydrophones. Blue triangles indicate positions of vocalizing beaked whales, e.g., the vocalization of Blainville's beaked whale (*Mesoplodon densirostris*) characteristically show acoustic energy above 20 kHz (Madsen et al. 2013). A cluster of beaked whale positions near hydrophones 3, 4 and 6 was used to locate the whales at the surface and collect eDNA for the third serial sample collection (see Table 1).

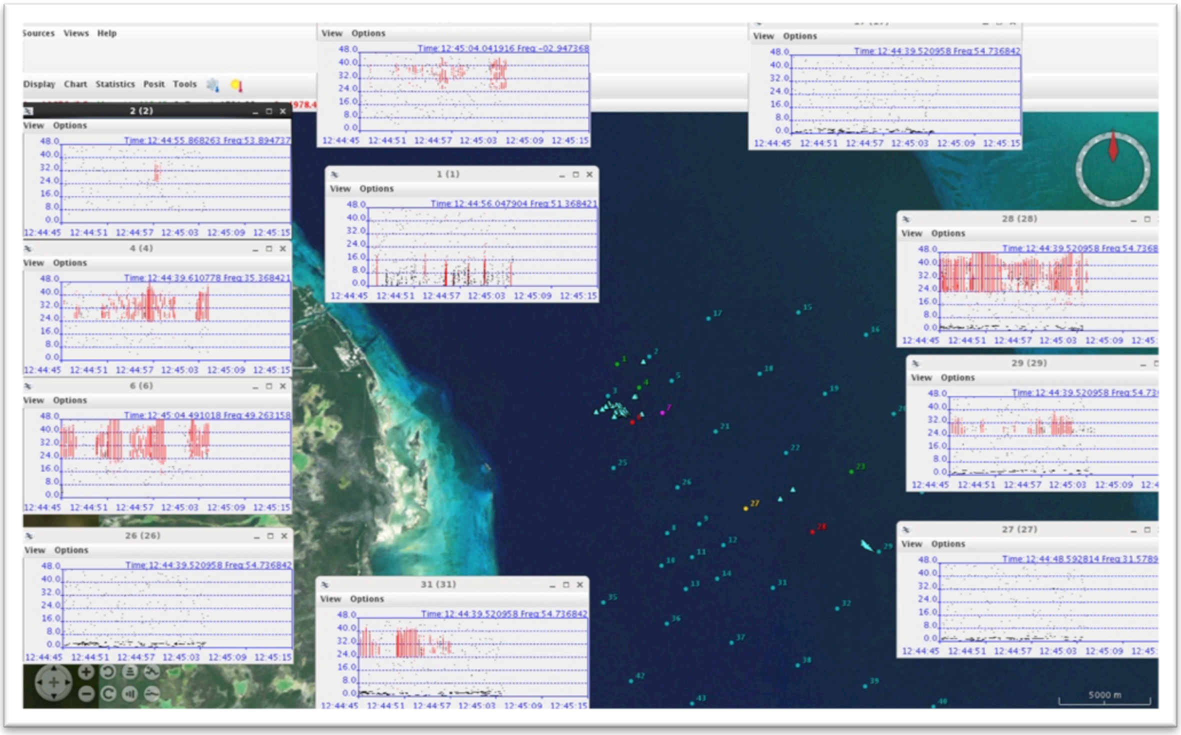

Supplement: S1 Fig — Acoustic activity is indicated by the color of the hydrophone, with red indicating highest activity. The insets show the time (x axis) and frequency in kHz (y axis) of vocalizations on selected hydrophones. Blue triangles indicate positions of vocalizing beaked whales, e.g., the vocalizations of Blainville’s beaked whale (Mesoplodon densirostris) characteristically show acoustic energy above 20 kHz [34]. A cluster of beaked whale positions near hydrophones 3, 4 and 6 was used to locate the whales at the surface and collect eDNA for the third serial sample collection (see Table 1). (PDF) [file pone.0291187.s001.pdf]
